# Where ketamine and dopamine collide

**DOI:** 10.7554/eLife.70148

**Published:** 2021-06-17

**Authors:** David J Marcus, Michael R Bruchas

**Affiliations:** 1 Department of Anesthesiology and Pain Medicine and Center for Neurobiology of Addiction, Pain, and Emotion, University of WashingtonSeattleUnited States; 2 Department of Anesthesiology and Pain Medicine, Center for Neurobiology of Addiction, Pain, and Emotion, and Department of Pharmacology, University of WashingtonSeattleUnited States

**Keywords:** dopamine, aversive learning, ketamine, mPFC, VTA, antidepressants, Mouse

## Abstract

Ketamine strengthens connections between two brain regions that are involved in the production and regulation of dopamine, which may explain how the drug can alleviate depression.

**Related research article** Wu M, Minkowicz S, Dumrongprechachan V, Hamilton P, Xiao L, Kozorovitskiy Y. 2021. Attenuated dopamine signaling after aversive learning is restored by ketamine to rescue escape actions. *eLife* **10**:e64041. doi: 10.7554/eLife.64041

1987 was a watershed year in the history of antidepressants, with Prozac being the first selective serotonin reuptake inhibitor drug to be approved for use in the US. Prozac and other drugs that limit the reuptake of neurotransmitters such as serotonin, norepinephrine and dopamine would dominate the market for the next three decades. These treatments were marked improvements over their predecessors, but it has become clear that they are not the silver bullet they were once touted to be: moderate efficacy, insensitive populations, and untenable side effects have all limited their clinical utility (Warden et al., 2007).

Enter Ketamine. First synthesized over 75 years ago, this small, unassuming compound had so far been relegated to veterinary clinics as a pet anesthetic, while also doubling as a club drug that could induce dissociation and euphoria. In the early 2000s, however, reports started to emerge suggesting that a single dose of ketamine could have profound and lasting antidepressant effects (Berman et al., 2000). After over a decade of research, the US Food and Drug Administration (FDA) finally approved ketamine, in the form of ‘esketamine’, for the treatment of depression (Carboni et al., 2021). However, the mechanisms that drive the antidepressant effects of ketamine are poorly understood: this is unusual for an FDA-approved drug, although not unheard of for molecules used to treat affective disorders such as depression. How can a compound used to anesthetize cats or induce a psychedelic-like high have clinical utility?

Dopamine, the so-called ‘pleasure or reward neuromodulator’, is indispensable for regulating responses to rewards such as delicious foods, sex or addictive substances; however, it has also been implicated in certain mood disorders (Berridge, 2018). In fact, the emergence of depression has been linked to disruption in the activity of the dopamine-producing neurons present in the ventral tegmental area (VTA) of the brain, but few studies have examined whether ketamine elicits its antidepressant effects by altering the activity of these cells (Hamon and Blier, 2013). Now, in eLife, Yevgenia Kozorovitskiy and colleagues at Northwestern University – including Mingzheng Wu as first author – report how ketamine can strengthen brain circuits that include the VTA (Wu et al., 2021).

The researchers used a ‘learned helplessness’ experimental mouse model which mimics the blunted behavioral or emotional reactions that are one of the hallmarks of depression (Bylsma et al., 2008). The rodents were repeatedly exposed to mild electric shocks to the foot that were impossible to escape: over time, they learn that it was pointless to try to avoid this stressor, and they froze rather than try to escape. Antidepressants – including ketamine, as Wu et al. now show – alleviate this helplessness and restore escape behaviors. The researchers had also genetically manipulated the rodents to introduce a bioengineered molecule that emits light when neurons become activated, with the change in fluorescence being used as a proxy for neuronal activity (Inoue, 2020). The experiments revealed that in mice with learned helplessness, the activity pattern of VTA neurons was abnormal: however, ketamine treatment could reverse this disruption (Figure 1).

**Figure 1.**
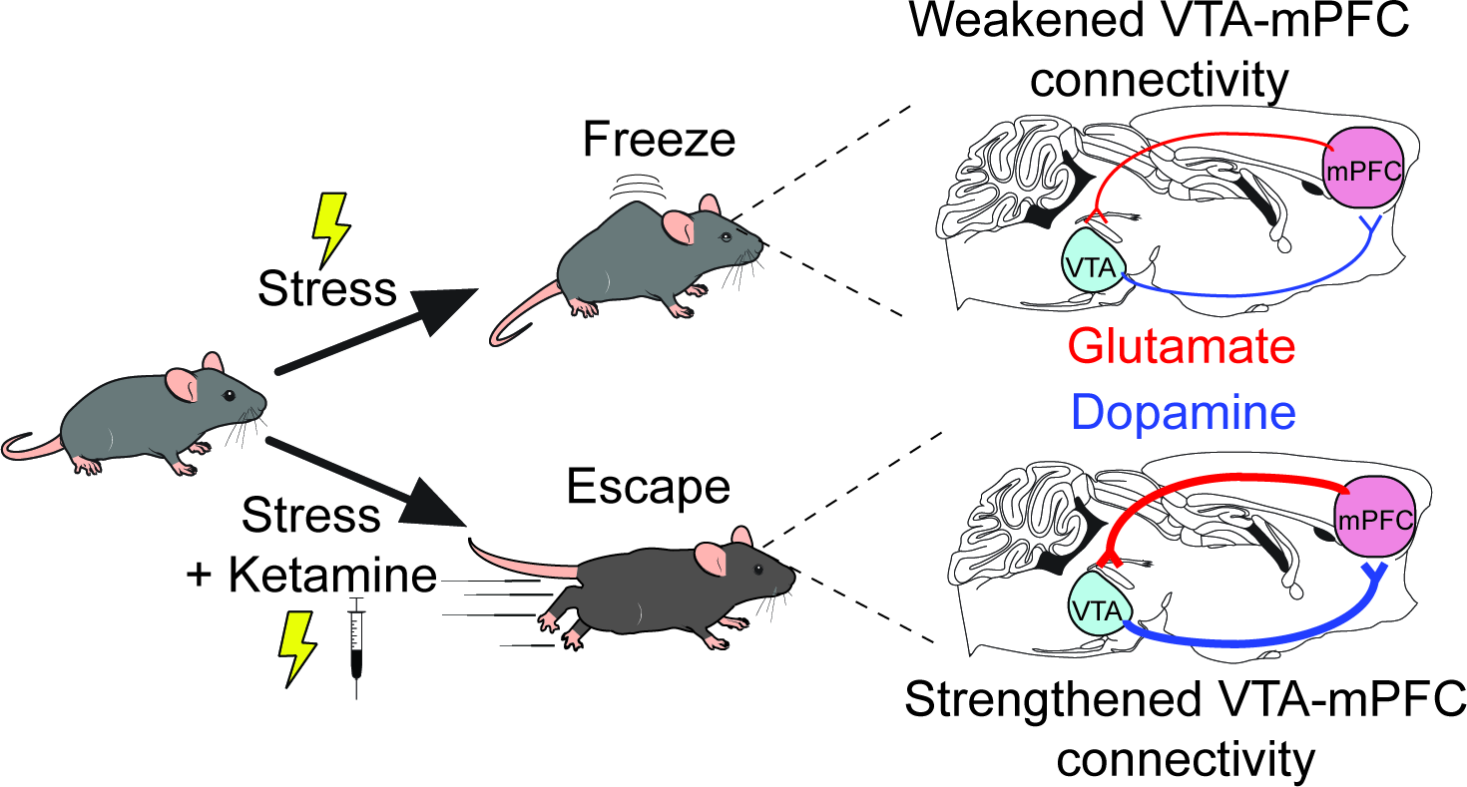
A new mechanism for the antidepressant effects of ketamine. Over time, mice that are exposed to repeated mild electric shocks from which it is not possible to escape stop trying to avoid this stressor; instead, they develop learned helplessness and ‘freeze’ rather than attempt to escape, a behavior reminiscent of the reduced behavioral responses observed in depression (top). This exposure to stress disrupts the activity of a circuit formed by dopamine-producing projections (blue lines) and glutamate-producing projections (red lines) that connect the ventral tegmental area (VTA) and the medial prefrontal cortex (mPFC). Receiving ketamine (bottom), however, has an antidepressant effect. The drug alleviates learned helplessness and allows the mice to display active coping behaviors such as trying to escape the stimuli, while also reversing the disruption in the circuits between VTA and mPFC.

These data clearly demonstrated that VTA dopamine neurons are involved in responding to stressors, but whether ketamine acts by changing the activity of the neurons themselves remained unresolved. To test for a causal link, Wu et al. selectively inhibited VTA neurons by using another bioengineered molecule, a receptor called DREADD that can only be selectively activated by a synthetic molecule (Dobrzanski and Kossut, 2017). Ketamine had no effect when the VTA neurons were inhibited, demonstrating that VTA dopamine neuron activity is necessary for the drug to have an antidepressant effect.

Further physiology experiments were then conducted in the VTA to determine how ketamine could impact neuronal activity, with, somewhat unexpectedly, few noticeable effects emerging. This suggested that rather than acting locally, ketamine most likely worked by modulating the activity of an upstream brain region that influences VTA activity. The medial prefrontal cortex (mPFC for short) represented an attractive target because it sends dense projections to the VTA, and because it mediates, in part, the antidepressant effects of katamine (Vertes, 2004; Moda-Sava et al., 2019).

Indeed, Wu et al. discovered that injecting ketamine specifically into the mPFC (but not other VTA-projecting brain regions) replicated the improvements observed when the mice were administered the drug systemically. In addition, antidepressant-like effects that mirrored those induced by ketamine emerged when mPFC neurons that express the receptor for dopamine were artificially activated using excitatory DREADDs.

The research presented by Wu et al. provides a unique understanding of the antidepressant effects of ketamine on canonical reward circuits. Specifically, the results suggest that the drug acts by strengthening a recurrent neural circuit between the VTA and the mPFC, which allows ketamine’s antidepressant properties to persist long after the compound has been cleared from the body. The data pave the way for future studies that directly examine how the effects of ketamine are mediated by mPFC projections to the VTA. Ultimately, this knowledge will help to find and design more selective compounds which can target these circuits to treat depression and related disorders.

## References

[bib1] Berman RM, Cappiello A, Anand A, Oren DA, Heninger GR, Charney DS, Krystal JH (2000). Antidepressant effects of ketamine in depressed patients. Biological Psychiatry.

[bib2] Berridge KC (2018). Evolving concepts of emotion and motivation. Frontiers in Psychology.

[bib3] Bylsma LM, Morris BH, Rottenberg J (2008). A meta-analysis of emotional reactivity in major depressive disorder. Clinical Psychology Review.

[bib4] Carboni E, Carta AR, Carboni E, Novelli A (2021). Repurposing ketamine in depression and related disorders: can this enigmatic drug achieve success?. Frontiers in Neuroscience.

[bib5] Dobrzanski G, Kossut M (2017). Application of the DREADD technique in biomedical brain research. Pharmacological Reports.

[bib6] Hamon M, Blier P (2013). Monoamine neurocircuitry in depression and strategies for new treatments. Progress in Neuro-Psychopharmacology and Biological Psychiatry.

[bib7] Inoue M (2020). Genetically encoded calcium indicators to probe complex brain circuit dynamics in vivo. Neuroscience Research.

[bib8] Moda-Sava RN, Murdock MH, Parekh PK, Fetcho RN, Huang BS, Huynh TN, Witztum J, Shaver DC, Rosenthal DL, Alway EJ, Lopez K, Meng Y, Nellissen L, Grosenick L, Milner TA, Deisseroth K, Bito H, Kasai H, Liston C (2019). Sustained rescue of prefrontal circuit dysfunction by antidepressant-induced spine formation. Science.

[bib9] Vertes RP (2004). Differential projections of the infralimbic and prelimbic cortex in the rat. Synapse.

[bib10] Warden D, Rush AJ, Trivedi MH, Fava M, Wisniewski SR (2007). The STAR*D project results: a comprehensive review of findings. Current Psychiatry Reports.

[bib11] Wu M, Minkowicz S, Dumrongprechachan V, Hamilton P, Xiao L, Kozorovitskiy Y (2021). Attenuated dopamine signaling after aversive learning is restored by ketamine to rescue escape actions. eLife.

